# Ultrasound Assessment in Merkel Cell Carcinoma: Case Report and Narrative Literature Review

**DOI:** 10.3390/reports9010043

**Published:** 2026-01-29

**Authors:** Vincenza Amoruso, Letizia Castelli, Anastasia Mercurio, Patrizia Matano, Giacomo Montaldi

**Affiliations:** 1 Recovery and Functional Reeducation Unit, Rehabilitation Department, Santa Corona Hospital, 17027 Pietra Ligure, Italy; 2 Musculoskeletal Ultrasound School, Italian Society for Ultrasound in Medicine and Biology, 40136 Bologna, Italy; 3 Department of Neurosciences, Università Cattolica del Sacro Cuore, 00168 Rome, Italy; 4 Unit of Anatomical Pathology, Department of Experimental Medicine, University La Sapienza, 00161 Rome, Italy; 5 Private Practice in Aesthetic Medicine, Center for Aesthetic Surgery and Aesthetic Medicine, 17031 Albenga, Italy

**Keywords:** Merkel cell carcinoma, ultrasound, skin cancer, radiologic imaging, early diagnosis, case report, dermatologic oncology

## Abstract

**Background and Clinical Significance** Merkel cell carcinoma (MCC) is a rare and aggressive neuroendocrine skin malignancy. Early diagnosis is essential to optimize therapeutic strategies and improve prognosis. However, the role of high-frequency ultrasound (HFUS) in the diagnostic and follow-up phases of MCC remains under-investigated and underutilized in clinical practice. **Case Presentation** We present a case of MCC initially referred to a physiatric outpatient clinic for a functional disorder of the third finger, where HFUS revealed a well-circumscribed, hypoechoic subdermal lesion with central and peripheral vascularity. Surgical excision, histopathology, and immunohistochemistry confirmed the diagnosis of Merkel cell carcinoma. The HFUS findings were correlated with histological features, and a structured sonographic follow-up protocol was established postoperatively. **Conclusions** This case highlights the diagnostic and prognostic potential of HFUS in MCC, especially in early detection, surgical planning, and longitudinal follow-up. A multidisciplinary approach integrating ultrasound imaging, surgery, and pathology may enhance diagnostic accuracy and patient management.

## 1. Introduction

Merkel cell carcinoma (MCC) is a rare and highly aggressive neuroendocrine malignancy of the skin, typically arising in sun-exposed areas of elderly or immunocompromised patients [1,2]. Its pathogenesis is primarily linked to the Merkel cell polyomavirus (MCPyV) or solar exposure with ultraviolet (UV)-induced mutations [3]. MCC is thought to arise or share a lineage with Merkel cells, specialized cutaneous mechanoreceptors that utilie PIEZO2 ion channels for tactile transduction [4]. Clinically, MCC often presents as a firm, painless, rapidly growing nodule. Due to its high propensity for local recurrence and early metastasis, prompt diagnosis is critical [5,6]. However, as the diagnosis is rarely made on clinical grounds alone, the gold standard remains histopathological examination with immunohistochemistry [2]. Imaging plays a crucial role in management of cutaneous neoplasms. While PET-CT and MRI are established modalities for staging and follow-up, the role of ultrasound (US) is frequently overlooked in current MCC guidelines. Ultrasound (US), although widely used in dermatology for melanomas, cutaneous lymphomas, and benign adnexal tumors, is rarely discussed in the context of MCC [7]. When reported, MCC often appears as a solid, hypoechoic mass with heterogeneous internal echoes and increased vascularity [8].

High-frequency ultrasound (HFUS) offers several potential advantages for the assessment of superficial lesions, including high spatial resolution and real-time imaging; however, its role is not systematically addressed in most current MCC imaging guidelines.

**Clinical Significance** 

This manuscript aims to address this diagnostic gap by presenting a clinical case supported by a narrative analysis of the imaging literature. This case underscores the valuable role of HFUS in the early recognition of MCC; specifically, it illustrates how HFUS can reveal atypical and suspicious features in lesions initially considered benign, prompting timely biopsy. We suggest that integrating HFUS into dermatologic workflows may enhance diagnostic suspicion, support targeted histological sampling, and provide a complementary, non-invasive tool for short-term lesion assessment.

While these findings suggest that HFUS may represent a practical adjunct in the multidisciplinary evaluation of MCC, these conclusions are based on single case and should be regarded as preliminary, requiring confirmation in larger, multicenter studies to validate its clinical utility.

## 2. Methods

**Figure 1:**
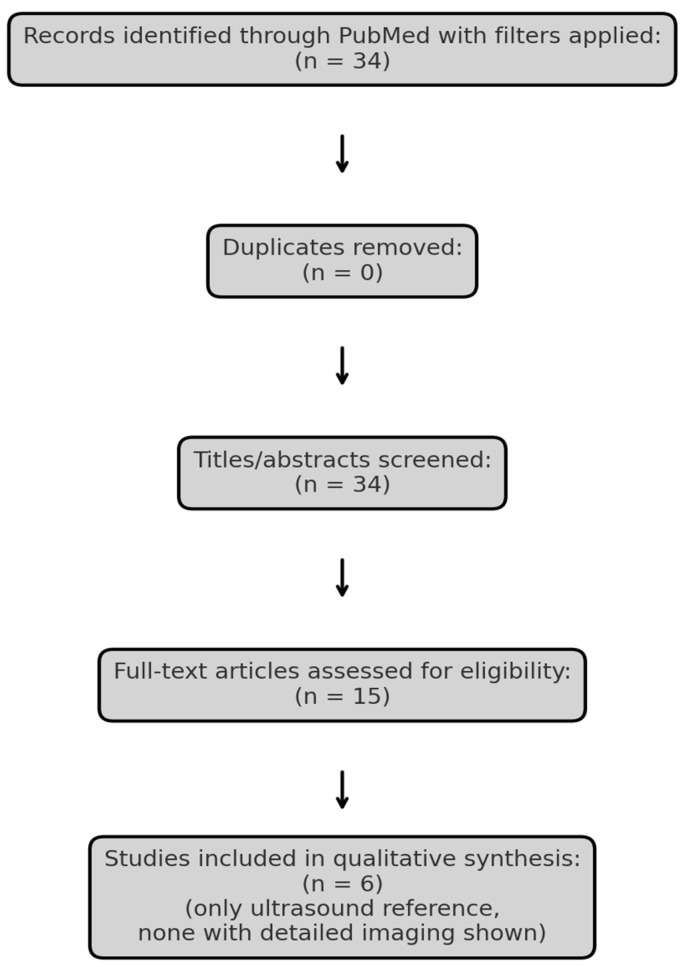
PRISMA flow diagram illustrating the selection process of studies included in the review.

To explore the current evidence, we conducted a narrative review using the PubMed database. The search strategy was defined using the terms “Merkel cell carcinoma” AND (“ultrasound” OR “MRI” OR “imaging”), with filters for language (English), species (Humans), publication type (Clinical Trial, Meta-analysis, Randomized Controlled Trial, Review, Systematic Review) and publication date (January 2015–April 2025). Literature screening was guided by the PRISMA (Preferred Reporting Items for Systematic Reviews and Meta-Analyses) framework exclusively for study identification and selection, without performing a formal systematic review or meta-analysis (Figure 1, Table 1). Studies lacking clinical imaging data or full-text availability were excluded. One additional, relevant article Hernández-Aragüés et al. (2017) [9] was manually added despite being classified as a “Letter,” as it represents the only published ultrasound-based case series on MCC. The nature of the available literature precluded formal study risk-of-bias assessment or quantitative data synthesis.

## 3. Case Presentation

**Patient Information and Clinical Findings** 

**Figure 2:**
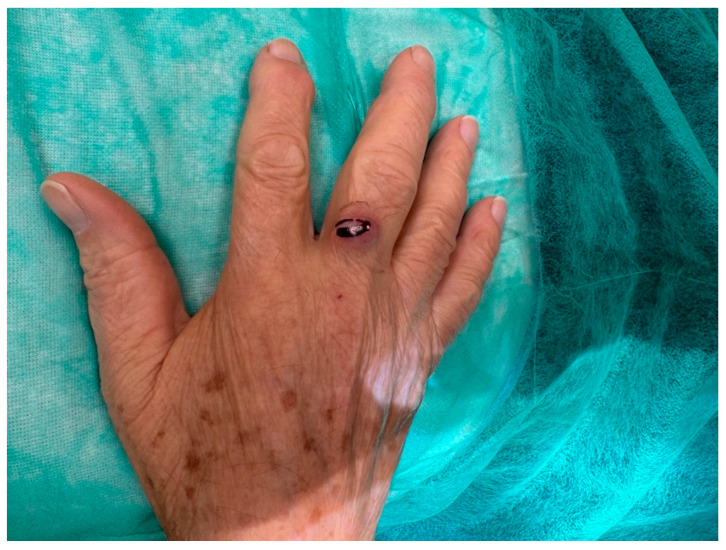
An 81-year-old right-handed Caucasian male presented to our department with a six-month history of a painless, solid, smooth, and fixed 1 × 1 × 0.8 cm nodule on the dorsal aspect of the proximal phalanx of the right middle.

An 81-year-old Caucasian male presented to our outpatient clinic with a firm, painless, and fixed subcutaneous nodule on the dorsal aspect of the right middle finger, measuring approximately 1 × 1 × 0.8 cm (Figure 2). The patient reported no history of trauma or systemic symptoms. His past medical history was unremarkable, with no known immunosuppressive conditions.

Although no formal history of chronic actinic damage or previous non-melanoma skin cancers was documented, the patient was an elderly fair-skinned male with a history of outdoor occupational and recreational activities, including agricultural work and long-term residence in a sun-exposed rural area, suggesting a possible cumulative ultraviolet exposure.

Due to the lesion’s long-standing stability and the absence of rapid growth or inflammatory signs, the initial clinical suspicion favored a benign condition, such as a cutaneous adnexal tumor or an inclusion cyst.

**Diagnostic Assessment**

A high-frequency ultrasound (HFUS) examination was performed using a 22 MHz linear probe. Ultrasonography revealed a well-circumscribed, hypoechoic, and homogeneous subdermal mass situated superficially to the extensor tendon of the third finger. The use of HFUS allowed for improved visualization compared to standard-frequency ultrasound (8–14 MHz), which would be expected to depict a nonspecific hypoechoic nodule with limited internal detail.

**Figure 3:**
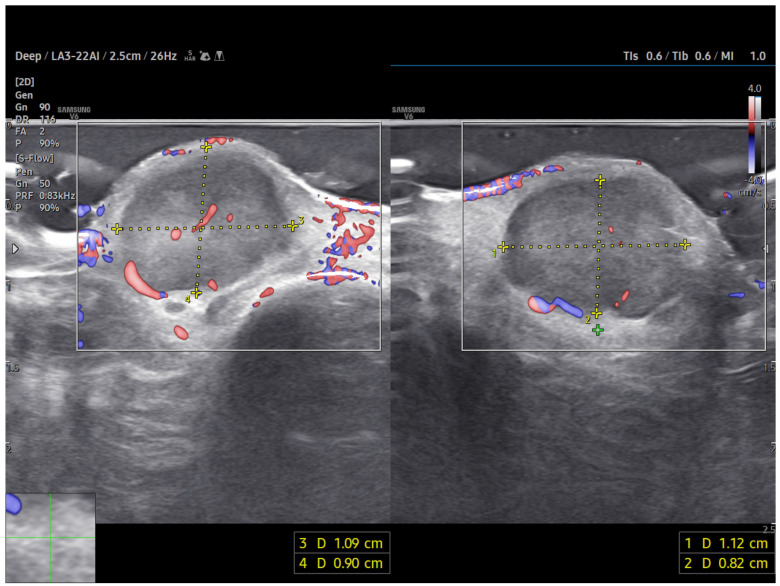
US imaging of MCC. The lesion appeared as a well-circumscribed, homogeneous, subdermal mass resting on the extensor tendon of the third finger on the dorsal ulnar side. On ultrasonography, the mass showed a finely heterogeneous echotexture with vascularization on power Doppler, both peripheral and central. One vascular pole appeared to supply the lesion via a central vessel.

Power Doppler imaging demonstrated both peripheral and central vascular signals, with a distinct vascular pole supplying the lesion through a central feeding vessel (Figure 3). The lesion’s well-defined margins, combined with a robust internal vascular pattern, shifted the clinical concern toward a possible malignancy.

**Therapeutic Intervention**

**Figure 4:**
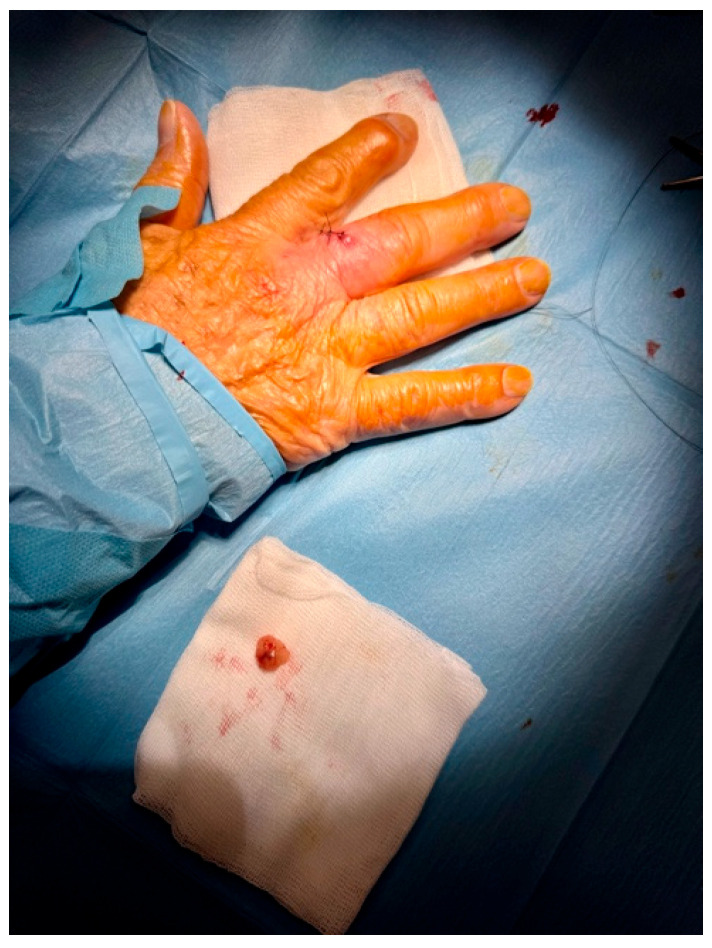
Excision with narrow margins was performed for accurate and reliable histopathological analysis. The lesion was excised with 3 mm peripheral margins and closed with simple suture under local anesthesia.

Based on the suspicious imaging findings, the patient underwent surgical excision under local anaesthesia. The mass was completely excised with minimal tissue disruption, and primary closure was achieved using simple interrupted sutures (Figure 4). The procedure was well tolerated and no intraoperative complications occurred.

**Histopathological Findings**

**Figure 5:**
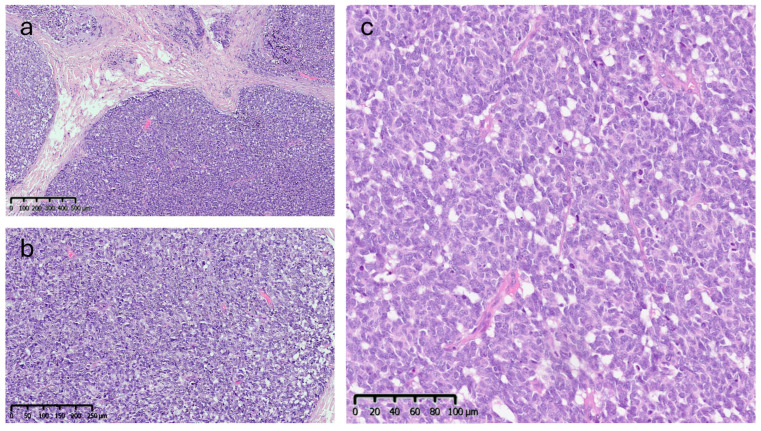
Histopathological features (Hematoxylin & Eosin): (**a**) low-power view (4×) showing a nodular growth pattern. (**b**,**c**) Higher magnification (10× and 20×) showing dense proliferation of medium-sized monomorphic cells with oval nuclei, scant cytoplasm, and finely granular chromatin.

Microscopic analysis revealed an epithelial proliferation characterized by a nodular growth pattern, with solid clusters of cells separated by fibrous septa and focal areas of central necrosis. Cytologically, the neoplastic cells appeared monomorphic and medium-sized, exhibiting oval nuclei, scant cytoplasm, and the typical finely granular chromatin. The mitotic index was 10/mm^2^ (Figure 5).

**Figure 6:**
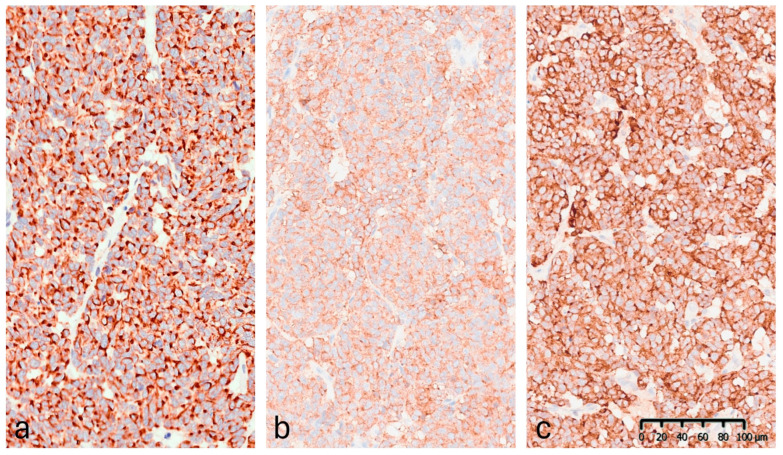
Immunohistochemical staining (20×): (**a**) CK20: positive with perinuclear dot-like staining pattern. (**b**) Synaptophysin: diffuse granular cytoplasmic expression. (**c**) Chromogranin A: strong and diffuse cytoplasmic positivity.

Immunohistochemistry (IHC) demonstrated diffuse cytoplasmic positivity for synaptophysin, chromogranin A, CAM5.2, and CK20, which displayed the characteristic dot-like perinuclear staining pattern (Figure 6). Conversely, the lesion tested negative for CK7, TTF-1, S100, and p63. Testing for MCPyV was not performed. The Ki-67 proliferation index was 35–40%. These findings confirmed the diagnosis of MCC. The lesion was completely excised.

**Follow-Up and Outcomes**

**Figure 7:**
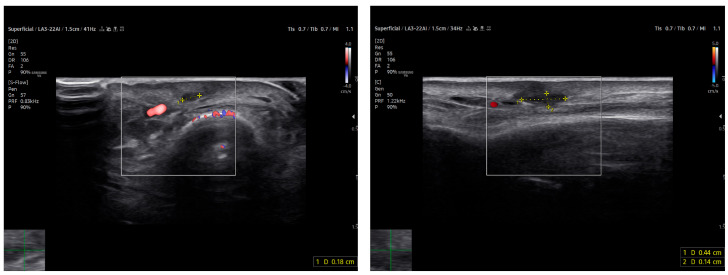
At follow-up, the ultrasound scan displays the area of the resected lesion and the scar region in both transverse (on the **left**) and longitudinal sections (on the **right**) above the long extensor tendon of the uninjured third finger.

**Figure 8:**
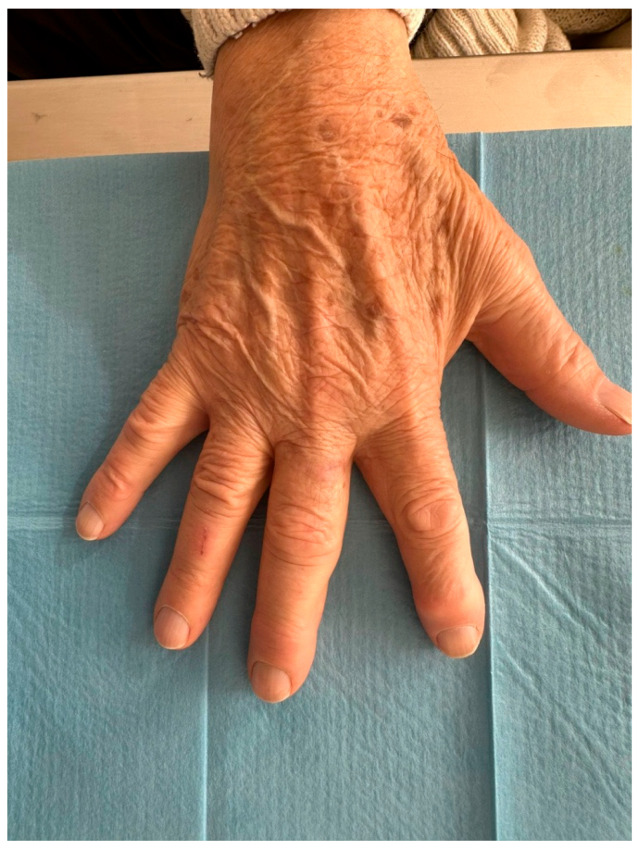
At follow-up, the patient maintained a wide range of motion without pain.

HFUS follow-up at three weeks post-excision showed a well-demarcated, avascular and hypoechoic area consistent with postoperative changes and scar tissue, with no evidence of local residual tumor (Figure 7). Clinically, the patient demonstrated excellent wound healing and retained a full, pain-free range of motion in the affected digit (Figure 8). This short-term HFUS follow-up was limited to postoperative site assessment only and does not allow any conclusions regarding long-term oncological control or recurrence surveillance.

**Ethical Considerations**

This case report describes a non-interventional observational study. Ethical approval was not required, as no experimental procedures or off-label treatments were performed. Written informed consent was obtained from the patient for publication of clinical data and images.

## 4. Discussion

Merkel cell carcinoma (MCC) is an aggressive neuroendocrine skin tumour with a rising incidence and a well-known propensity for early regional and distant spread. Early diagnosis remains challenging due to its variable clinical presentation and the rarity of the disease, particularly in digital locations. Imaging plays a crucial role in the diagnostic process, yet current literature shows a striking paucity of high-quality sonographic documentation regarding MCC.

Our narrative review highlights that very few publications include robust or high-frequency sonographic descriptions of MCC [16]. Notably, Hernández-Aragüés et al. described ultrasound findings in seven cases, observing hypoechogenicity and increased vascularity; however, that report lacked standardized terminology and longitudinal follow-up data [9]. Despite following the PRISMA framework for literature screening, this review has limitations: the use of a single database (PubMed) may have excluded pertinent studies indexed elsewhere or published in languages other than English. Furthermore, the findings derived from our clinical case are preliminary and, given the inherent limitations of case reports, require validation in larger cohorts.

Although MCC does not exhibit a unique, pathognomonic ultrasound pattern, it commonly presents as a solid, hypoechoic mass with heterogeneous internal echoes and prominent vascularity. Occasionally, distinctive ‘plume-of-smoke’ linear bands or thick septae may be observed [8]. Other relevant findings include posterior acoustic enhancement and epidermal thickening. In our case, the combination of marked hypoechogenicity and prominent, often central, vascularity was strongly suggestive of malignancy. This imaging pattern may assist in differentiating MCC from common benign nodular lesions, such as epidermal inclusion cysts, fibromas, or ganglion cysts, which typically demonstrate absent or minimal internal vascularity.

As such, the internal vascular pattern observed may represent a useful imaging clue for early clinical suspicion.

Magnetic resonance imaging (MRI) remains the preferred modality for soft-tissue characterisation [6], while ^18F-FDG PET-CT is the standard for detecting nodal and distant metastasis [14]. However, HFUS provides unique advantages for superficial and digital lesions, offering real-time evaluation of lesion borders, involvement of adjacent structures, and precise depth measurement. By providing complementary information to cross-sectional imaging, HFUS may support earlier suspicion and inform surgical planning. As emerging technological refinements, such as ultra-high-frequency probes, become more widely available, sonography may evolve into a more central tool for evaluating suspicious nodules in dermatologic oncology [17]. The present case contributes to the field by providing a structured clinicopathological comparison supported by 22 MHz high-resolution imaging, while acknowledging that true imaging–pathology correlation is limited by the spatial resolution of the probe used.

HFUS guided the decision to proceed with excision and, in the immediate postoperative phase, confirmed the absence of residual tumour by identifying an avascular, well-defined scar. However, we must emphasize that the assessment period in this report was limited to three weeks. This brief window reflects only the immediate postoperative outcome and does not provide sufficient evidence regarding long-term oncological control or recurrence surveillance.

Overall, beyond its diagnostic value, ultrasound represents a cost-effective, accessible, and radiation-free modality suitable for integration into routine dermatologic workflows. Its application is expanding across dermatologic oncology, including melanoma, cutaneous lymphomas, adnexal tumours, and inflammatory dermatoses [17]. For MCC specifically, our findings suggest that ultrasound may assist in surgical margin planning, guide-targeted biopsies, and enable close surveillance. These features are of particular importance in high-risk tumours located in anatomically delicate sites, such as the digits, where tissue sparing is crucial.

This report underscores the potential of HFUS as a practical adjunct to established techniques, contributing to improved clinical decision-making while highlighting the need for systematic sonographic documentation in rare cutaneous malignancies.

## 5. Conclusions

Merkel cell carcinoma remains a diagnostic challenge, and this case suggests that high-frequency ultrasound may support earlier clinical suspicion in selected superficial lesions, while requiring further validation in larger, prospective studies. Its integration into the standard diagnostic work-up may significantly contribute to better lesion characterization, more precise procedural planning, and non-invasive longitudinal follow-up.

## Figures and Tables

**Table 1:** Summary of the six studies included in the narrative review, selected based on relevance to radiologic imaging in Merkel cell carcinoma. While PET/CT and MRI are commonly discussed, none of the studies provided original ultrasound imaging, highlighting the lack of sonographic documentation in the current literature.

Author (Year)	Title	Imaging Modalities	Key Findings
**Akaike et al. (2019) [10]**	Imaging of Merkel Cell Carcinoma: What Imaging Experts Should Know	CT, MRI, PET/CT, Ultrasound (general reference)	Comprehensive overview of MCC imaging techniques, limited reference to ultrasound.
**Llombart et al. (2017) [11]**	Merkel Cell Carcinoma: An Update of Key Imaging Techniques, Prognostic Factors, Treatment, and Follow-up	MRI, CT, PET/CT (no US images)	Discusses diagnostic performance of various modalities, no imaging figures.
**Grandhaye et al. (2015) [12]**	Focus on Merkel cell carcinoma: diagnosis and staging	Radiographs, MRI, PET/CT	Emphasizes staging utility of radiology, no sonographic detail.
**Lebbé et al. (2015) [13]**	Diagnosis and treatment of Merkel Cell Carcinoma. European consensus-based interdisciplinary guideline	CT, MRI, PET/CT (review guideline)	Consensus guideline with general imaging recommendations.
**Shim & Kim (2022) [14]**	Diagnostic Test Accuracy of 18F-FDG PET or PET/CT in Merkel Cell Carcinoma	PET/CT	PET/CT offers high sensitivity for MCC staging.
**Cassler et al. (2016) [15]**	Merkel Cell Carcinoma Therapeutic Update	Overview (minimal imaging detail)	Mentions imaging role in management, no modality-specific focus.

## Data Availability

The original contributions presented in this study are included in the article. Further inquiries can be directed to the corresponding author.

## Abbreviations

The following abbreviations are used in this manuscript:

MCC	Merkel cell carcinoma
HFUS	High-frequency ultrasound
US	Ultrasound
MRI	Magnetic resonance imaging
PET-CT	Positron emission tomography–computed tomography
CK20	Cytokeratin 20
H&E	Hematoxylin and Eosin
PRISMA	Preferred Reporting Items for Systematic Reviews and Meta-Analyses
